# Pulmonary bacterial pathogens in cystic fibrosis patients and antibiotic therapy: a tool for the health workers

**DOI:** 10.1186/1755-7682-1-24

**Published:** 2008-11-07

**Authors:** Henrique Douglas M Coutinho, Vivyanne S Falcão-Silva, Gregório Fernandes Gonçalves

**Affiliations:** 1 Laboratório de Pesquisa em Produtos Naturais, Departamento de Ciências físicas e Biológicas, Centro de Ciências Biológicas e da Saúde, Universidade Regional do Cariri, Crato (CE), Brazil; 2 Laboratorio de Genética de Microrganismos, Departamento de Biologia Molecular, Centro de Ciências Exatas e da Natureza, Universidade Federal da Paraíba, João Pessoa (PB), Brazil

## Abstract

Cystic fibrosis is the most common and best known genetic disease involving a defect in transepithelial Cl- transport by mutations in the CF gene on chromosome 7, which codes for the cystic fibrosis transmembrane conductance regulator protein (CFTR). The most serious symptoms are observed in the lungs, augmenting the risk of bacterial infection. The objective of this review was to describe the bacterial pathogens colonizing patients with cystic fibrosis. A systematic search was conducted using the international bibliographic databanks SCIELO, HIGHWIRE, PUBMED, SCIRUS and LILACS to provide a useful and practical review for healthcare workers to make them aware of these microorganisms. Today, *B. cepacia*, *P. aeruginosa *and *S. aureus *are the most important infectious agents in cystic fibrosis patients. However, healthcare professionals must pay attention to emerging infectious agents in these patients, because they represent a potentially serious future problem. Therefore, these pathogens should be pointed out as a risk to these patients, and hospitals all over the world must be prepared to detect and combat these bacteria.

## Introduction

Cystic fibrosis (CF) is the most common autosomal genetic disease in North America, affecting 1:2000 Caucasian individuals [1]. This disease is caused by mutations affecting the cystic fibrosis conductance regulator protein (CFTR) and is characterized by chronic lung malfunction, pancreatic insufficiency and high levels of chloride in sweat. Its high mortality index is evident when lung and spleen are affected, but other organs can also be affected. The persons affected die by progressive bronchiectasis and chronic respiratory insufficiency [2,3]. This disease affects persons without distinction of age or sex but can be asymptomatic in a great number of cases [3]. Failure of innate defense mechanisms and the lack of mucocilliary clearance in the airways stimulate primary and recurrent bacterial infections, blockage of airways, inflammation and chronic bacterial infections [4,5].

During the first decade of life of CF patients, *Staphylococcus aureus *and *Hemophilus influenzae *are the most common bacteria isolated from the sputum, but in the second and third decade of life, *Pseudomonas aeruginosa *is the prevalent bacteria. In Germany, analysis of the sputum from CF patients during a period of 12 months showed the presence of *P. aeruginosa *in 50% of these individuals, *S. aureus *in 63.3%, *Haemophilus influenzae *in 16.6%, *Stenotrophomonas maltophilia *in 15% and nontuberculous Mycobacteria (NTM) in 13.3% [6].

Due to this succession of bacterial populations in CF patients and due to the importance of these pathogens in prognosis, the objective of this article was to review and identify known and emerging bacterial pathogens associated with pulmonary problems and involved with cystic fibrosis. For this objective, a systematic search was conducted using the international bibliographic databanks SCIELO, HIGHWIRE, PUBMED, SCIRUS and LILACS. The uniterms Cystic fibrosis, infection and antibiotic therapy were used in a retrospective search between 1990 to 2007. Any articles with this theme, reporting bacterial pathogen associated with CF patients with no distinction of sex and age were selected and only the articles describing pathogens and the antibiotic therapy were really used.

### Bacterial pathogens associated with pulmonary risk

#### Major pathogens

##### Mycobacterium sp

The nontuberculous Mycobacteria (NTM) are a group of microorganisms that is very common in chronic pulmonary diseases. The increase in the life expectancy of CF patients has also increased the prevalence of *Mycobacteria *in the CF population [7]. The clinical impact of these microorganisms in CF patients is unclear, because Oliver *et al*. [8] found that CF patients infected with NTM, observed for 15 months, did not show a decline in respiratory function. These microorganisms were isolated from older CF patients, all of them with perfect respiratory function, and were associated with a high frequency of *S. aureus *and a low one of *P. aeruginosa *when compared with patients without NTM, indicating that the presence of these bacteria may be taken as a good prognostic sign [9].

The most common NTM infecting CF patients are *Mycobacterium abscessus*, *Mycobacterium avium*, and *Mycobacterium intracellulare *[6], but Sermet-Gaudelus *et al*. [10] identified other NTM from CF patients, including *M. fortuitum*, *M. gordonae *and *M. kansasii*. Today, the NTM more likely associated with the disease is *Mycobacterium abscessus *[11]. The identification of the causal species of NTM is essential and requires genetic techniques [12]. Treatment depends on the mycobacterial species. For *M. avium*, combined therapy with rifampicin, clarythromycin and ethambutol must be extended 12 months after negativation. M. abscessus infection is particularly resistant to therapy. Usual treatment is a one month course of intravenous imipenem or cefoxitin plus amikacin followed by oral clarithromicin plus ethambutol for at least 12 months after negativation. In case of local lesions, surgery is an option [12].

##### Staphylococcus aureus

Usually, this is the first pathogen to infect and colonize the airways of CF patients, being the most common pathogen [13]. This microorganism is prevalent in children and may cause epithelial damage, opening the way to the adherence of other pathogens such as *Pseudomonas aeruginosa *[14]. However, other studies indicate that *S. aureus *is a co-infective pathogen associated with *P. aeruginosa*. Together, the inflammatory process is more intense due the additive effect of these two pathogens [15]. Before the use of antibiotics in the treatment of infections, *S. aureus *was the causative agent of several deaths in children with CF. Today, this risk is not so serious, but CF patients not given the correct antibiotic therapy show a higher prevalence of *S. aureus *in the nasal epithelium when compared to treated patients [16]. About the prevalence of this pathogen, the same strain of *S. aureus *remains in the patient for 1–2 years [17].

Methicillin-resistant *S. aureus *(MRSA) has become a major nosocomial pathogen with a progressive increase in prevalence also in CF populations. The acquisition of MRSA occurred only in adulthood [18]. In Europe, the spread of MRSA varies widely among centers, ranging from 5 to 14% [19]. MRSA is a major pathogen in the hospital setting causing serious infections that usually present multiresistance to many antibiotics. Moreover, the increased frequency of this organism in the community, especially with carriage of virulence factors, including the presence of the virulence marker *pvl*, is a matter of concern [20,21].

Small colony variants (SCVs) of *S. aureus *constitute a bacterial population with distinctive phenotypic traits of *S. aureus *populations from CF patients [22]. These populations are involved with the colonization of older patients [23], but Sadowska *et al*. [24] isolated these strains from children between 1.5 and 9 years old with a SVR prevalence of 31.7%.

##### Pseudomonas aeruginosa

*P. aeruginosa *is an oxidase-positive Gram-negative motile rod [25]. Vonberg & Gastmeier [26] showed that this bacterium colonizes CF patients in more than 50% of cases. This bacterium is a part of the normal microbial population of the respiratory tract, where it is an opportunistic pathogen in CF patients. This is more prevalent in adult CF patients, as infection has been shown in 20% CF patients 0–2 years old while in 81% in adult groups (>18 years old) [27]. Aaron *et al*. [28] showed that all CF patients with chronic infections and older than 16 years are infected with *P. aeruginosa*, but Burns *et al*. [29] found that 97.5% of children had *P. aeruginosa*. The capacity of this bacterium to develop biofilm is a characteristic that allows it to survive for very long periods in the lungs of CF patients [30].

Isolated from *P. aeruginosa *can be differentiated in terms of its morphotypes, including mucoid, not mucoid and those with biofilm, which vary their patterns of susceptibility to antibiotics. This differentiation causes several problems in the treatment, because is necessary identify the morphotype to choose the treatment strategy [31,32].

##### Burkholderia ssp

*Burkholderia cepacia *complex (BCC) is a complex of Gram-negative rod, aerobic, mesophilic and chemoorganotrophic [33]. This is a bacterial complex with nine genomic species (genomovars) [34,35]: genomovar I (*B. cepacia*), II (*B. multivorans*), III (*B. cenocepacia*), IV (*B. stabilis*), V (*B. vietnamiensis*), VI (*B. dolosa*), VII (*B. ambifaria*), VIII (*B. anthina*), IX (*B. pyrrocinia*) [35,36].

Infected CF patients show high levels of BCC in the salivary fluid, indicating the possibility of indirect transmission by kissing and sexual contact [36], but the transmission rates, prognosis and mortality are distinctly characteristic for each genomovar, as the treatment strategies[33,37]. Because the difficulties in the culture and identification of genomovar, this is one of the most important opportunistic bacterial pathogens of CF patients [38,39]. Other bacteria the same genus *Burkholderia*, as *Burkholderia gladioli *and *Burkholderia pseudomallei*, which are distinct from the *Burkholderia cepacia *complex have also been reported in patients with cystic fibrosis [40-42]. Members of *B. cepacia *complex are very resistant to antibiotic therapy because its genome is very plastic and suffers several mutations and adapts itself, making it a hard challenge for treatment. Its resistance is mainly due the production of enzymes with capacity to inactivate the substances used in the treatment [43]. By this fact., the accuracy and fast detection of this bacterium are essential to evaluate risks, prognostics and epidemiology of cystic fibrosis [35].

### Minor pathogens

#### Achromobacter xylosoxidans

This bacterium is a Gram-negative rod, anaerobic, motile, oxidase and catalase positive and lactose non-fermentative. It is usually distributed in the environment, but can be a human pathogen causing bacteremia, meningitis and pneumonia [44]. This is a pathogen with a growing incidence in CF patients and a high coinfection rate with *P. aeruginosa *[45,46].

#### Inquilinus limosus

Coenye *et al*. [47] in 2002 isolated 8 strains from airway secretions of CF patients in the United States, that were identified as a new genus called *Inquilinus*, belonging to α-proteobacteria and further identified as *I. limosus*. This bacterium is a mesophilic Gram-negative rod, non-spore forming. Due to its recent characterization, we have little knowledge about its natural habitat, prevalence and pathogenicity, but CF patients infected with this bacterium have been identified in hospitals in France, Spain and Germany [48,49].

#### Ralstonia sp

These bacteria are Gram-negative and non-fermentative rods, and little is known about the natural occurrence and the pathogenicity of bacteria from the genus *Ralstonia*, mainly due to their difficult identification, where they are usually misidentified as *P. fluorescens *or a member of the *Burkholderia cepacia *complex [50-54].

Reports indicate a low prevalence of pathogen from this genus in CF patients, but Coenye *et al*. [52] showed the permanence of this pathogen in the sputum of CF patients for more than 20 months.

#### Pandoraea apista

This is a Gram-negative and non-fermentative bacterium that over the years has shown a growing isolation frequency among CF patients, representing a possible emerging pathogen in these patients [55,56]. Atkinson *et al*. [57] analyzed sputum cultures from 2 adult CF patients (30 and 36 years old, respectively), and found both colonized by this bacteria and coinfected with *P. aeruginosa*. This finding is very important due to the fact that these patients are first infected with *P. aeruginosa*, indicating that the latter pathogen may act as a starting point for *P. apista *infection.

#### Streptococcus pneumoniae

This microorganism is considered a transient pathogen in CF patients [58], mainly isolated from young CF patients [59]. The incidence is 5.5% in CF patients 12 and younger, but in children without the disease the frequency is 50% [60].

#### Stenotrophomonos maltophilia

This is a Gram-negative and non-fermentative rod that is frequently isolated from hospitals [61,62]. *S. maltophilia *is a pathogen of CF patients with a very constant incidence [63]. Goss et al. [62] observed that patients with *S. maltophilia *were older, showing a high rate of prior co-infection with *P. aeruginosa *and *B. cepacia*, but the prevalence of this pathogen in CF patients has been growing in the last years [64].

#### Haemophilus influenzae

This bacterium usually infects younger CF patients. In Brazil, 20.4% of CF children between 6 and 12 years old are infected by *H. influenzae *[65]. This bacterium undergoes hyper-mutation, which can be related to its resistance to antibiotics, making treatment more difficult [66].

#### Bordetella bronchiseptica

This is a Gram-negative coccobacillus, non spore-forming, strictly anaerobic, and catalase and coagulase positive [67]. This bacterium is part of the microbiota of the upper respiratory tract of many animals [68]. Magalhães *et al*. [67], reported the presence of it in a 27- year-old CF patient associated with *S. aureus*, which can be a potential zoonotic infectious agent, aggravating the CF patient situation.

### Treatment

Cystic fibrosis is characterized by chronic pulmonary infection with acute pulmonary exacerbation (APEs), where antibiotic therapy is necessary against opportunistic infections [69].

Previous studies have indicated that the presence of mucoidal *P. aeruginosa *was the most important risk factor for pulmonary deterioration [70,71]. By this fact, several article indicating method to control de colonizing pathogens in CF patients use *P. aeruginosa *as a microbial marker.

Gentamicin and tobramycin are recognized as standard antibiotics for the treatment of CF patients infected with *Pseudomonas aeruginosa*. Mulheran *et al*. [72] observed a higher utilization of gentamicin and tobramycin by pediatric patients and adults respectively. However, the authors make note of the greater cochleotoxic risk associated with gentamicin. Depending on the administration and dose used, tobramycin can be more or less efficient [73]. When this drug was used in a liposomal formulation and delivered as an aerosol, the drug bioavailability in pulmonary tissue and its effectiveness enhance [74,75].

Tests with animals have shown the augmentation of the amykacin concentration in the lung against *Pseudomonas aeruginosa*, when the drug is administered by ultrasonic nebulization or intravenously, but these levels decrease after the second administration [76].

Antibiotic combinations against *P aeruginosa*, such as the use of polymyxins combined with a β-lactamic are useful in antipseudomonial therapy, as shown in the work of Dong & Chung-Dar [77].

Azithromycin displays interesting therapeutic results in the treatment of CF patients infected with *P aeruginosa*. Wagner *et al*. [78] reported that azithromycin inhibits 80% of protein synthesis in *P aeruginosa *PAO1, affecting bacterial growth and the expression/exportation of products that stimulates the immune system such as pyocyanin.

Other point of discussion is the objective of the treatment of *P aeruginosa *infection: total eradication, using heavy doses of antibiotics with adverse symptoms, or the management of the infection, with a higher risk to develop the resistance? Few years ago, the eradication of chronic *P. aeruginosa *infection was considered impossible [79], but Ho *et al*. [80] e Pitt *et al*. [81] showed that new populations of *P. aeruginosa *(after eradication) were different of the first ones and more sensitive to the antibiotics, showing that persistent populations of *P. aeruginosa *in the airway would increase the antibiotic resistance with time because of prolonged exposure to antibiotics, as in the case of management, indicating the eradication as the most interesting strategy.

For other microorganisms such as *B. cepacia*, commonly resistant to several antimicrobial drugs used by CF patients, the better treatment choice is a drug combination. Combinations of two antibiotics from different classes such as meropenem-minocycline, meropenem-amikacin and meropenem-ceftazidime or three different antibiotics such as tobramycin, meropene and an additional antibiotic were more effective than the use of any antibiotic alone [78]. Similar results were observed against *P. aeruginosa *by Dong *et al*. [77] who showed that the better treatment is the combination of meropenem/tobramicin or ceftazidime/tobramicin.

However, new therapeutic perspectives are needed, such as from the work of Zhang *et al*. [82] who evaluated the *in vitro *effectiveness of 150 antimicrobial peptides in multidrug resistant strains of *P. aeruginosa, Stenotrophomonas maltophilia, Achromobacter xylosoxidans *and *S. aureus*. A better activity was observed for several peptides compared to most of the antibiotics used in the clinic. Similar results were obtained by Etienne *et al*. [83] who used defensins and observed a drastic reduction in bacterial growth.

The quality of life and life expectancy of CF patients have improved considerably as a result of the control of bronchopulmonary bacterial colonization and acute infectious exacerbations [82-85].

These reports indicate the necessity for more research into the discovery and rational design of new antibacterial drugs that will be more efficient in combating infections in cystic fibrosis patients. However, the use must be well defined. Our search indicates that the combination of 2 or more antibiotics may represent an interesting alternative in the CF treatment, colonized for any bacterial pathogen.

Other point of interest is the indication of aerosolized and biofilm-inhibitory drugs may control and avoid the colonization of the respiratory tract by several pathogens cited in this study. Maybe, using this several approaches, we will maximize the control of the colonizers and the infections that affect the CF patients.

## Conclusion

Several factors affect transmission, such as the type of bacterial strain, the immune state of the patient and the use of contaminated medical equipment. Therefore, all CF patients infected or colonized the major pathogens cited in this article must be isolated in a single room because they represent sources for nosocomial transmission of the microorganism to other patients during the treatment [17,55].

Although the epidemiology of bacterial pathogens in CF patients has become more complex, the life expectancy of these patients continues to increase. This has led to a better control of the transmission of these pathogens by the separation of adults and children with CF in different treatment centers. Furthermore, the utilization of basic preventive guidelines (hand washing and use of masks, gloves and protectors), combined with disinfection techniques to be applied at home or hospital make control easier. These precautions help reduce the impact of infections in CF patients. In addition, educational programs to support administrative measures, guidelines for the control of nosocomial infections and the assistance to healthcare workers and to the families of the patients to show the importance of these measures are essential tools for blocking the transmission of these bacterial pathogens to CF patients.

## Competing interests

The authors declare that they have no competing interests.

## Authors' contributions

VSFS and GFG contributed to conception and design, designed the review, carried out the literature research, and manuscript preparation. HDMC contributed to conception and design, carried out the manuscript editing and manuscript review. All authors read and approved the final manuscript.
